# Near-miss Thoracic Spine Solitary Plasmacytoma with Neurological Deficit during Pregnancy: A Case Report

**DOI:** 10.5704/MOJ.2211.022

**Published:** 2022-11

**Authors:** KH Teh, J Thilak, HS Lim, AA Yahaya, ZA Kamarul-Bahrin

**Affiliations:** 1 Department of Orthopaedics, Hospital Seberang Jaya, Permatang Pauh, Malaysia; 2 Department of Orthopaedics, Hospital Pulau Pinang, George Town, Malaysia

**Keywords:** plasmacytoma, solitary spinal tumour, pregnancy, spine surgery

## Abstract

Solitary plasmacytoma (SPC) account for only 5% of plasma cell neoplasms, and the literature hardly reports spinal SPC with a neurological deficit. Furthermore, spinal surgical intervention during pregnancy is rarely encountered and often requires multidisciplinary collaboration and management. The objective of this case report is to highlight this near-miss diagnosis and spinal surgical intervention during pregnancy. A 31-year-old woman with 24 weeks gestation presented with sudden paralysis and incontinence, with an underlying history of chronic backpain over a two-month period. Initially, she was treated for musculoskeletal back pain by obstetric colleagues during an antenatal visit, and no radiograph was performed. A non-contrasted spinal MRI was eventually requested when she started to show bilateral lower limb weakness, numbness and incontinence. The MRI highlighted thoracic vertebrae T11 vertebra plana with kyphotic deformity and a paraspinal soft tissue mass compressing the spinal cord causing spinal cord oedema. Our initial working diagnosis was spinal tuberculosis (TB), considering TB is highly endemic in Malaysia. However, TB workup was negative, and we proceeded with spinal surgery and transpedicular biopsy. Neurology improved significantly after surgery. Eventually, serum protein electrophoresis reported plasma dyscrasia, and HPE confirmed plasmacytoma. The patient was referred to a haematologist for steroidal and chemotherapy treatment.

## Introduction

Plasmacytoma is a rare, malignant, proliferative disease, accounting for only 5% of plasma cell neoplasms^1^. Plasmacytoma is subdivided into solitary plasmacytoma (SPC) and extramedullary plasmacytoma^1^. SPC mainly affects the axial skeleton and is scarcely reported in the literature^1^. Until now, there has been no uniform consensus about prognostic factors and treatment choice due to the rarity of the disease^1^. Moreover, both radio-imaging workup and spinal surgery during pregnancy invariably present a major concern among orthopaedic personnel due to the rarity of the condition. Multidisciplinary team management is definitely needed in this case.

We hereby report a case of near-miss thoracic vertebrae SPC in a pregnant woman who manifested sudden paraplegia, incontinence and chronic back pain, with special emphasis on its workout towards diagnosis, surgical management and outcome throughout her pregnancy.

## Case Reports

**Fig. 1.**
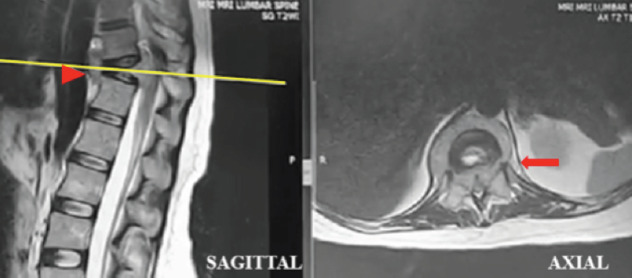
Magnetic resonance imaging (MRI) of the patient’s spine. Arrowhead shows T11 vertebra plana with kyphotic deformity. Arrow demonstrates paravertebral soft tissue mass formation extending into spinal canal causing spinal canal stenosis and cord compression.

**Fig. 2.**
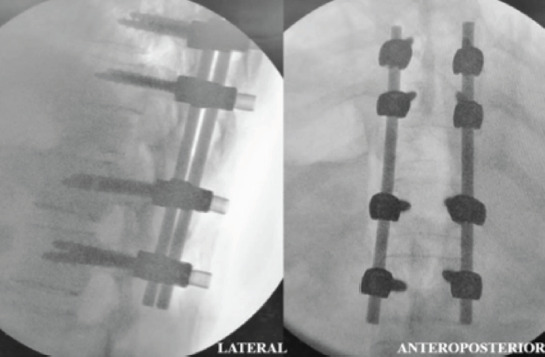
Intra-operative spinal image intensifier (II) view after posterior instrumentation. All pedicle screws are well positioned and there is no scoliotic or kyphotic deformity.

**Fig. 3.**
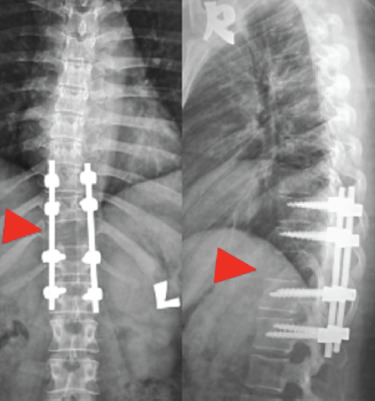
Spinal radiograph eight months after surgery demonstrates T11 vertebrae body radiolucency (arrowhead). Implants are intact, with no other vertebrae involvement.

A 31-year-old pregnant woman, at 24 weeks gestation, complained of back pain over a two-month period. She had significant instability pain and night pain without radicular pain. Initially, she was treated for musculoskeletal pain by an obstetrician and no spine radiograph was performed. However, she started showing bilateral lower limb numbness and weakness after falling down in a sitting position one week ago. Further examination revealed no notable history of infection, TB, malignancy or traumatic injury. Clinically she looks pink and has no lymphadenopathy or organomegaly. Initial neurological examination on admission noted a T12 neurological level of ASIA C; however, this worsened to ASIA B one day after admission. Plain MRI (Fig. 1) revealed T11 vertebrae plana with a paravertebral soft tissue mass causing cord compression. Routine blood investigations, peripheral blood film and both infective and tuberculosis screening were all normal. As such, posterior instrumentation, laminectomy and transpedicular biopsy were performed under controlled image intensifier (II) guidance (Fig. 2). Slime materials encountered from the biopsy tract tested negative for TB culture. Serum protein electrophoresis eventually reported plasma cell dyscrasia, and a histopathological exam (HPE) demonstrated features suggestive of plasmacytoma. The patient was started on both chemotherapy and steroidal treatment by a haematologist. Eight months after surgery, the patient has no back pain and near-normal recovered neurology, ASIA D. She is able to walk independently without a walking aid. Radiography (Fig. 3) reveals the intact implant with good spine alignment; however, the T11 vertebra body remains radiolucent. We plan for anterior reconstruction once chemotherapy treatment is completed.

## Discussion

SPC of the spine during pregnancy is rare and has never been reported in the literature^1^. This situation is rarely encountered; thus, our initial working diagnosis was TB of the spine, considering inflated TB is endemic in our country, the young age of the patient, typical MRI presentation (vertebrae destruction and paravertebral soft tissue mass), and a slightly elevated erythrocyte sedimentation rate. However, the medical team insisted on intra-operative biopsy before starting anti-TB therapy. Spinal SPC was eventually established by delayed serum protein electrophoresis and intra-operative HPE. We learned that even in cases where radiographic features are highly suspicious of TB, spine biopsy and HPE is mandatory, especially when the clinical picture and other TB workups are negative.

We did posterior instrumentation and decompression on top of the biopsy because the patient’s bilateral lower limb numbness and weakness started just one week before admission, and power had progressively worsened to complete zero a day after admission. Thus, considering the worsening neurology, significant instability pain and HPE will take weeks to get reported, decompression with instrumentation was conducted after detailed discussion with the patient. Furthermore, her neurology improved from ASIA B to ASIA C immediately after surgery.

Spinal surgery in pregnant women is infrequent but should not be denied in case of acute spinal compression, to prevent irreversible permanent neurological damage and significant lifetime disability. The American College of Obstetricians and Gynaecologists (ACOG)^2^ recommend that a pregnant woman should never be denied indicated surgery, regardless of trimester. However, elective surgery should ideally be performed in the second trimester, when preterm contractions and spontaneous abortion are least likely, or should be postponed until after delivery^2^.

Diagnostic chest and spine radiography in pregnancy is safe. No single radio-imaging study will exceed the minimal foetal radiation dose (50mGy) to cause possible spontaneous miscarriage, congenital anomaly, mental retardation or microcephaly at any gestational stage^3^. Moreover, lead shielding can reduce foetus radiation exposure by up to 50%^3^. Intra-operative fluoroscopy is unavoidable in spine surgery. Our strategy to limit image intensifier (II) radiation exposure is using a bony landmark to determine surgical level before the first scan, proper aiming of the II device before scanning and, above all, to avoid unnecessary scans. Generally, radiation exposure of a single II scan ranges from 0.9 to 1mGy^3^. A total of 13 II scans were made in our case, accounting for 13mGy, far below the level of concern (50mGy). We prefer non-contrasted MRI, considering that gadolinium may cross the placenta and induce nephrogenic systemic fibrosis in the child^4^.

Concern regarding teratogenesis of anaesthetic agents and their usage in pregnancy is redundant^5^. However, anaesthetic agents can cause potential neonatal respiratory depression and should only be administered when necessary, during maternal surgery. Thus, the neonatal team should be available in such situations to provide respiratory support^5^. Literature review demonstrates no historic causal relationship between non-obstetric surgery and either miscarriage or preterm birth^5^. If the foetus is pre-viable (<26 weeks’ gestation), the Doppler ultrasound scan to ascertain foetal heart rate before or after surgery is sufficient^2^. In our case, the patient had regular foetal Doppler ultrasound checked and monitored for foetal heart rate by her obstetric team, per shift throughout admission, both before and after surgery.

In conclusion, spinal surgery during pregnancy is safe provided extra precautions are taken and should not be denied if clearly indicated. This is essential to avoid delayed diagnosis and unnecessary morbidity and mortality.
